# The effect of combined transcranial direct current stimulation and peripheral nerve electrical stimulation on corticospinal excitability

**DOI:** 10.1371/journal.pone.0214592

**Published:** 2019-03-29

**Authors:** Shota Tsuiki, Ryoki Sasaki, Shota Miyaguchi, Sho Kojima, Kei Saito, Yasuto Inukai, Mitsuhiro Masaki, Naofumi Otsuru, Hideaki Onishi

**Affiliations:** 1 Graduate School, Niigata University of Health and Welfare, Niigata city, Japan; 2 Institute for Human Movement and Medical Sciences, Niigata University of Health and Welfare, Niigata city, Japan; 3 Rehabilitation Center of Shiobara Hot Spring Hospital, Tochigi Medical Association, Tochigi, Japan; BG-Universitatsklinikum Bergmannsheil, Ruhr-Universitat Bochum, GERMANY

## Abstract

Transcranial direct current stimulation (tDCS) and peripheral nerve electrical stimulation (PES) can change corticospinal excitability. tDCS can be used to non-invasively modulate the cerebral cortex’s excitability by applying weak current to an electrode attached to the head, and the effect varies with the electrode’s polarity. Previous studies have reported the effect of combined tDCS and PES on corticospinal excitability; when compared to single stimulation, combined stimulation increases cortical excitability. In contrast, another study reported that the effect of tDCS is attenuated by PES; hence, there is no consensus opinion on the effect on combined stimulation. Therefore, this study aimed to clarify the effect of combined tDCS and PES on corticospinal excitability. In Experiment 1, the combined stimulation of anodal tDCS and PES (anodal tDCS + PES) was performed, and in Experiment 2, a combined stimulation with PES, after cathodal tDCS (PES after cathodal tDCS), was performed using a homeostatic metaplasticity theoretical model. In Experiment 1, anodal tDCS produced a significant increase from baseline in motor-evoked potential (MEP) amplitude 10 min after stimulation, but no significant changes in MEP amplitude were observed with PES or the anodal tDCS + PES condition. Experiment 2 showed a significant decrease in MEP amplitude immediately after cathodal tDCS, and a significant increase in MEP amplitude 15 min after PES, but no significant change in MEP amplitude was observed with sequential PES following cathodal tDCS. In conclusion, our data indicate that PES with anodal tDCS suppressed the effect of tDCS. Also, PES after cathodal tDCS did not induce homeostatic metaplasticity and increase corticospinal excitability.

## Introduction

Transcranial direct current stimulation (tDCS), a noninvasive electrical stimulation method inducing excitatory changes in the corticospinal circuitry [1], can be used to modulate the cerebral cortex’s excitability by applying weak current to an electrode attached to the head. Cortical excitability increases under the anodal electrode, and decreases under the cathodal electrode, by interposing tDCS for 5 min at an intensity of 1 mA in the primary motor cortex (M1); this effect lasts several minutes [2]. Regarding the mechanism of the effect of tDCS, polar changes in neurons’ resting membrane potential have been reported [3], and the activation of cortical *N*-methyl-D-aspartate (NMDA) receptors has been verified pharmacologically [4, 5]. A recent study has reported that, in astrocytes, synaptic transmission is likely to be enhanced by increased intracellular Ca^2+^ concentrations [6]. However, significant inter-individual variability in response to tDCS has been reported in healthy individuals [7, 8].

Peripheral electrical stimulation (PES) can induce excitatory changes in the corticospinal circuitry [9–12]. In animal experiments, corticospinal excitability significantly increased when electric stimulation was applied for 2 h to rats’ sciatic nerves [13]. Human studies have reported that PES also increases corticospinal excitability significantly [10, 14]. In addition, the effect of electrical stimulation varies depending on the stimulation intensity. For example, corticospinal excitability decreases when stimulation at the sensory threshold intensity is given, but it increases when stimulation at the motor threshold intensity is given [9, 11, 14]. However, reports also indicate that, even with stimulation at the motor threshold intensity, corticospinal excitability does not change with continuous electrical stimulation, increasing only with intermittent PES which repeated stimulation and rest [9]. The mechanism increasing the corticospinal excitability result from PES depresses the gamma-aminobutyric acid system suppressor in M1 [15].

Recently, the combined stimulation of tDCS and PES effects on corticospinal excitability and motor performance have been studied [16–19]. A previous report has shown that, when anodal tDCS is combined with PES, the post-intervention corticospinal excitability duration is significantly longer than with anodal tDCS alone [17]. However, another study has demonstrated that post-intervention corticospinal excitability did not significantly change with combined anodal tDCS and PES [18]. Several aspects of these differences remain unclear, including whether the differences are due to stimulation conditions, homeostatic metaplasticity, or other gating mechanisms. In addition, the influence of combined tDCS and PES on corticospinal excitability remains unknown.

We aimed to clarify whether it is possible to increase corticospinal excitability by a combination of tDCS and PES. In Experiment 1, we examined the effect of 10 min of anodal tDCS and PES, simultaneously delivered, on corticospinal excitability. Consistent with gating theory [20], we assumed that corticospinal excitability would be further increased by applying anodal tDCS, which reduces intracortical suppression [21] and increases excitability of M1, at the same time as PES. This theory proposes that gating of the plastic response occurs because of a higher net calcium influx and stronger NMDA receptor-dependent post-synaptic response [20] related to diminished inhibition or increased facilitation, upstream from cortical output neurons [20, 22].

Conversely, it is possible to reduce the threshold for synaptic plasticity induction by decreasing corticospinal excitability [20, 23]. Previous studies have shown that corticospinal excitability increased when high frequency, repetitive transcranial magnetic stimulation was provided after cathodal tDCS which reduces corticospinal excitability [24]. Thus, we based Experiment 2 on homeostatic metaplasticity theory. Homeostatic metaplasticity is thought to decrease the threshold for inducing synaptic plasticity by lowering the neuronal activity in M1 before the intervention [20]. We hypothesized that corticospinal excitability would further increase with the intervention of PES after cathodal tDCS of M1. We examined the effect of PES on corticospinal excitability when it immediately succeeded cathodal tDCS. We hypothesized that corticospinal excitability would increase with the gating mechanism in Experiment 1 and homeostatic metaplasticity in Experiment 2.

Transcranial magnetic stimulation (TMS) was used to evaluate corticospinal excitability. TMS can be used to noninvasively stimulate M1, allowing motor-evoked potentials (MEPs) to be recorded from the target muscle [25–27]. MEP is a method to record surface electromyograms by stimulating M1 [25–27]. The values of MEP amplitude elicited via TMS reflect the magnitude of corticospinal excitability. It is thus possible to evaluate changes in the magnitude of corticospinal excitability by comparing the values of MEP amplitude before and after the intervention.

## Materials and methods

### Experimental conditions

In this study, Experiments 1 and 2 were set up and performed using three experimental groups. In Experiment 1, the groups included: anodal tDCS of the left M1 (the anodal tDCS condition), PES of the right ulnar nerve (the PES condition), and simultaneous anodal tDCS and PES (the anodal tDCS + PES condition). In Experiment 2, the groups included: cathodal tDCS of the left M1 (the cathodal tDCS condition), PES of the right ulnar nerve (the PES condition), and PES after cathodal tDCS (PES after cathodal tDCS condition).

### Subjects

In Experiment 1, 15 healthy subjects aged 21.1 ± 0.6 years (mean ± standard deviation) participated; four were female and 13 were right-handed. In Experiment 2, 15 healthy subjects aged 22.3 ± 4.0 years (mean ± standard deviation) participated; two were female and 11 were right-handed. None of the subjects was taking medications or had a history of physical, neurological, or psychiatric disorders. We fully explained the research protocol, and all subjects gave their written informed consent to participate. This study was approved by the ethics committee at the Niigata University of Health and Welfare, Niigata, Japan. In all experiments, subjects were seated in a comfortable reclining chair with a mounted headrest, with their right forearm placed on the table during experiments.

### Electromyography recording

Surface electromyography (EMG) was recorded from the right first dorsal interosseous muscle (FDI), via disposable Ag/AgCl electrodes, in a belly-tendon arrangement. The earth electrode was wrapped around the right forearm. The EMG signals were amplified (×100) by an amplifier (A-DL-720-140, 4 Assist, Tokyo, Japan), filtered (high pass, 20 Hz), digitized at 4 kHz using an A/D converter (Power Lab 8/30, AD Instruments, Colorado Springs, CO, USA), and stored on a lab computer for later offline analysis (LabChart7, AD Instruments).

### Motor-evoked potential (MEP) recording

MEPs were used to evaluate corticospinal excitability before and after each intervention. A Magstim 200 (Magstim, Dyfed, UK) was used as a magnetic stimulator, and a figure-of-eight TMS coil (diameter, 9.5 cm) was placed tangentially at approximately 45° from the midline, with the handle facing posterolateral to the subject’s skull. The optimal coil position over the left M1 region for each subject was defined as the site eliciting the largest MEP (hot spot). The coil’s position and orientation for the hot spot were marked according to magnetic resonance imaging via Visor2 TMS Neuronavigation (eemagine Medical Imaging Solutions GmbH, Berlin, Germany). The TMS intensity used was the lowest pre-intervention stimulus intensity that induced a MEP with a 1 mV peak-to-peak amplitude in the relaxed FDI muscle. The magnetic stimulation interval was set to 4 to 6 seconds.

### tDCS

tDCS was delivered using a direct current stimulator (Eldith, NeuroConn GmbH, Germany) through a pair of saline-soaked surface sponge electrodes (5 × 7 cm, 35 cm^2^). In Experiment 1, the anodal electrode was placed at the left M1, and the cathodal electrode was placed above the contralateral orbit. The current intensity was 2 mA (current density, 0.057 mA/cm^2^) [28]. In Experiment 2, the cathodal electrode was placed at the left M1, and the anodal electrode was placed above the contralateral orbit. The current intensity was 1 mA (current density, 0.028 mA/cm^2^) [8, 29]. For both Experiments 1 and 2, tDCS was applied for 10 min (fade-in/fade-out time, 5 s), and the electrode attached to the left M1 was placed on the left scalp over the hot spot identified by TMS. For sham stimulation in Experiment 2, the cathodal tDCS was turned on for 30 s [30]. All the conditions other than the time of stimulation were the same for the cathodal tDCS condition in Experiment 2.

### PES

PES was delivered through bar electrodes to the right ulnar nerve at the wrist using an electrical generator (SEN-8203, Nihon Kohden, Tokyo, Japan). The electrical stimulation was delivered using a square wave with a pulse duration of 0.2 ms. Current was delivered at 30 Hz, and the stimulus intensity was determined to be 110% of the motor threshold at which the minimum stimulus intensity elicited M-waves. In addition, the stimulus pattern of 4 sec on, 6 sec off was used for repetitive stimulation and rest [9–11]. In Experiment 1, the stimulus duration was set to 10 min, which is the same stimulation duration, used for anodal tDCS to avoid overhang in the stimulation period during simultaneous stimulation. In Experiment 2, it was set to 20 min in accordance with the study by Chipchase et al. [9].

### Experimental procedures

**Fig 1:**
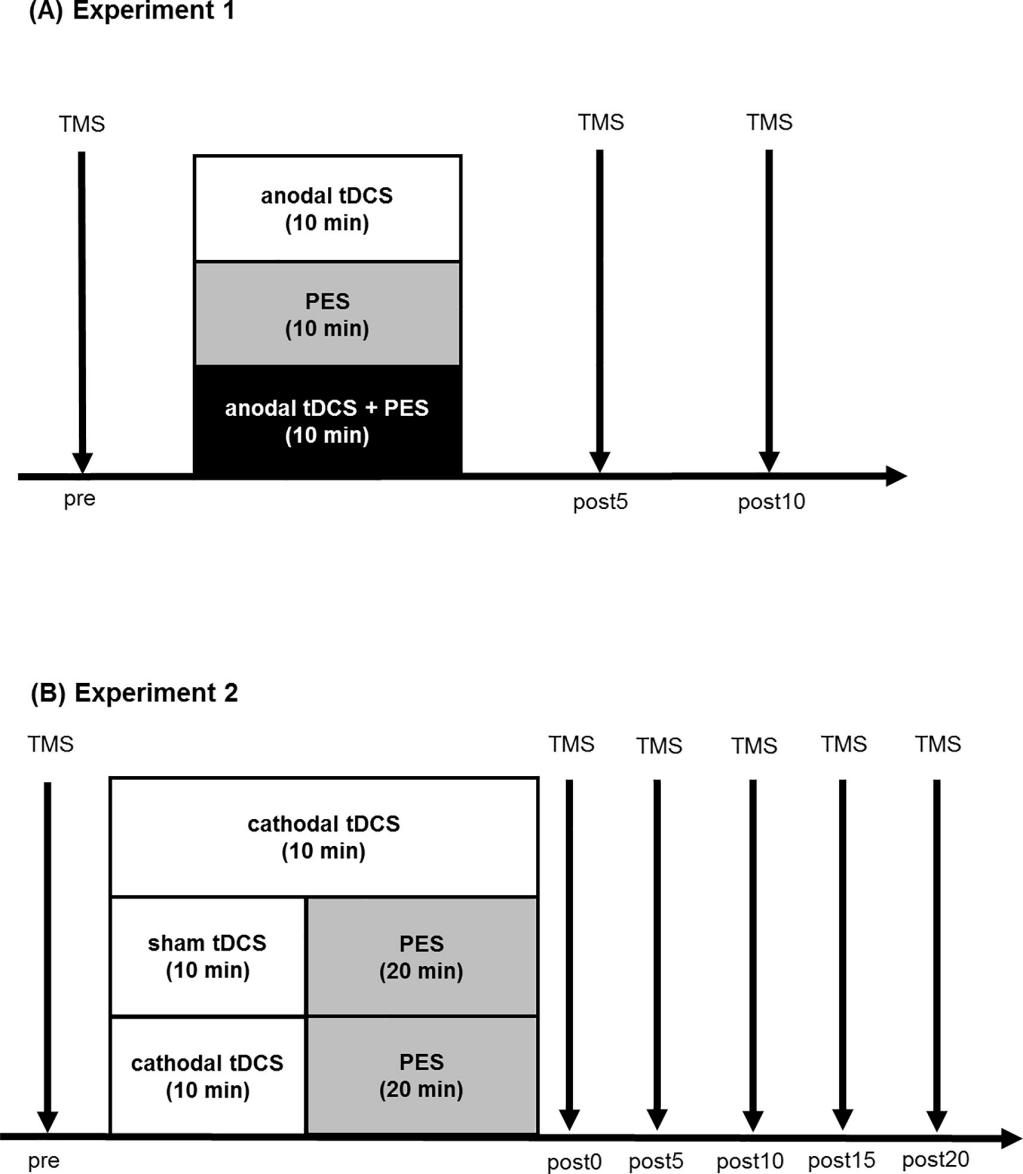
Outlines of the experimental procedures and timelines. In Experiment 1, subjects participated in three experimental conditions (anodal tDCS, PES, and anodal tDCS + PES). MEPs induced by TMS were measured before intervention (pre), 5 min (post5) and 10 min (post10) after intervention. In Experiment 2, the subjects participated in three experimental conditions (cathodal tDCS, PES, and PES after cathodal tDCS). MEPs induced by TMS were measured before intervention (pre), immediately (post0), 5 min (post5), 10 min (post10), 15 min (post15), and 20 min (post20) after intervention. In both experiments, each stimulation condition was spaced out over at least 72 hours and randomized.

The experimental procedures are shown in **Fig 1**. In Experiment 1, 24 MEPs were measured using TMS before (pre), 5 min (post5), and 10 min (post10) after the intervention. The same TMS intensity was used before and after the interventions. In Experiment 1, the three interventions (anodal tDCS, PES, and anodal tDCS + PES) were applied to the same subject in random order, spaced by at least 72 h.

Experiment 2 also had three interventions (cathodal tDCS, PES, and PES after cathodal tDCS) for the same subjects. 15 MEPs were measured before, just after, and every 5 min after the intervention for 20 min (post0, post5, post10, post15, and post20) by TMS. Interventions were randomly spaced by least 72 h.

TMS was applied between 12 and 25 times, in accordance with previously published methods [28, 31–33].

### Data analysis

LabChart software (LabChart 7, AD Instruments) was used to analyze the MEPs. In Experiment 1, the peak-to-peak amplitudes of 22 of the 24 recorded MEPs (excluding the maximum and minimum) were averaged for each time point (pre, post5, post10).

In Experiment 2, the peak-to-peak amplitudes of 13 of the 15 recorded MEPs (excluding the maximum and minimum) were averaged for each time point (pre, post0, post5, post10, post15, post20).

### Statistical analysis

Statistical analyses were performed using SPSS 21.0 for Windows (IBM, Armonk, NY, USA). In Experiments 1 and 2, Dunnett’s test of multiple comparisons was used. Statistical significance was set at *P* < 0.05.

## Results

### Experiment 1

1. Changes in MEP amplitudes before and after the interventions

**Fig 2:**
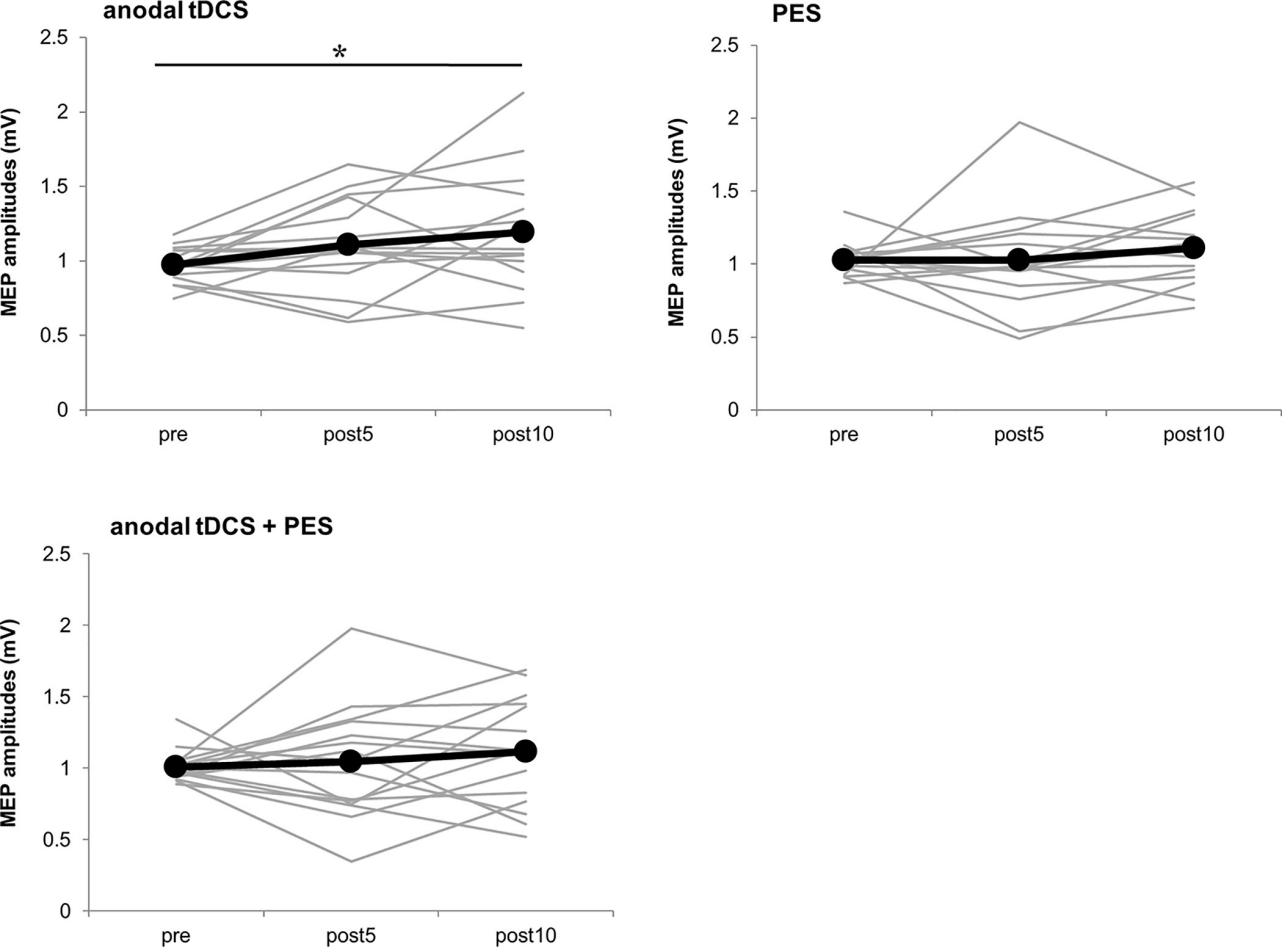
Time course of changes in MEP amplitudes for all subjects in Experiment 1. The mean MEP amplitudes, with anodal tDCS, PES, and anodal tDCS + PES, are shown before intervention (pre), 5 min (post5) and 10 min (post10) after intervention. In the anodal tDCS condition, MEP amplitudes significantly increased at post10 compared with pre (*P* < 0.05). In the PES condition, and the anodal tDCS + PES condition, no significant changes in MEP amplitudes were observed before or after the intervention. The gray line indicates the amplitude of the MEP for each individual. The black line indicates the mean amplitude of the MEP. The asterisk indicates a significant difference in MEP amplitudes compared to pre (*P < 0.05, Dunnett's test).

Changes in the time course of MEP amplitudes are shown in **Fig 2 and Table 1**. In the anodal tDCS condition, multiple comparisons showed a significant increase in MEP amplitude at post10 compared with pre (*P* < 0.05). However, in the PES and anodal tDCS + PES conditions, multiple comparisons test revealed no significant differences.

**Table 1:** Mean values of MEP amplitudes (mean ± standard error of the mean) before and after the three interventions in Experiment 1.

	pre	post5	post10
Anodal tDCS	0.97 ± 0.03	1.11 ± 0.08	1.19 ± 0.11*
PES	1.03 ± 0.03	1.03± 0.09	1.11± 0.07
Anodal tDCS + PES	1.01 ± 0.03	1.05± 0.10	1.12± 0.10

mean ± standard error (mV)

*P < 0.05 vs pre

### Experiment 2

**Fig 3:**
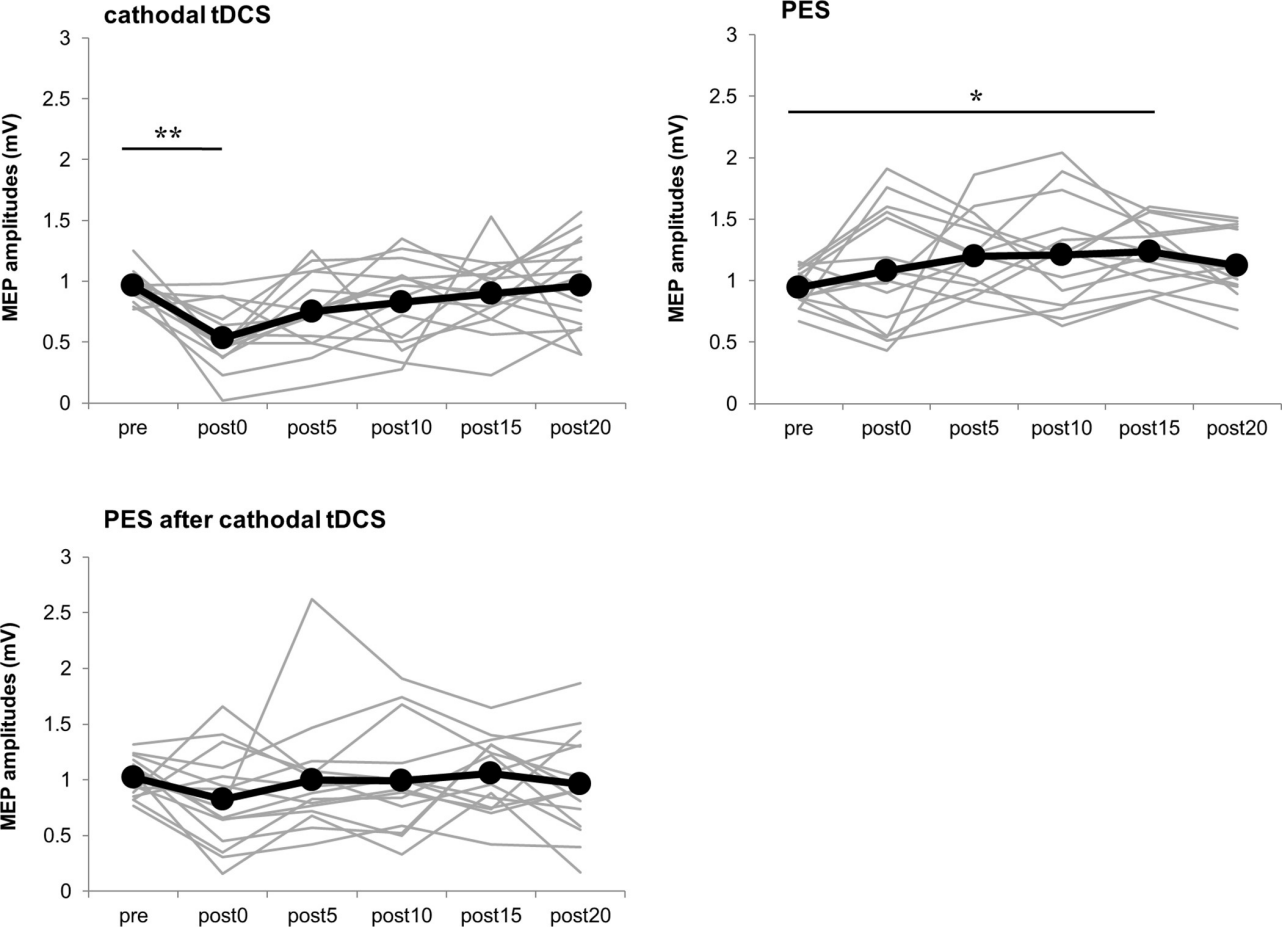
Time course of changes in MEP amplitudes for all subjects in Experiment 2. The mean MEP amplitudes at pre, immediately after the intervention (post0), and 5 min (post5), 10 min (post10), 15 min (post15), and 20 min (post20) after the intervention are shown. In the cathodal tDCS condition, the MEP amplitude significantly decreased at post0 compared to pre (*P* < 0.01). In the PES condition, the MEP amplitude significantly increased at post15 compared to pre (*P* < 0.05). In the PES after cathodal tDCS condition, no significant changes in MEP amplitudes were observed pre- and post-intervention. The gray line indicates the amplitude of the MEP for each individual. The black line indicates the mean amplitudes of the MEP. The asterisks indicate a significant difference of MEP amplitudes compared to pre (*P < 0.05, **P < 0.01, Dunnett's test).

Changes in the time course of MEP amplitudes are shown in **Fig 3 and Table 2**. In the cathodal tDCS condition, multiple comparisons showed a significant decrease in MEP amplitude at post0 compared to pre (*P* < 0.01). In the PES condition, the multiple comparison showed a significant increase in MEP amplitude at post15 compared to pre (*P* < 0.05). However, in PES after the cathodal tDCS condition, the multiple comparisons revealed no significance.

**Table 2:** Mean values of MEP amplitudes (mean ± standard error) before and after the three interventions in Experiment 2.

	pre	post0	post5	post10	post15	post20
Cathodal tDCS	0.96 ± 0.03	0.53 ± 0.07**	0.75 ± 0.08	0.83 ± 0.09	0.9 ± 0.08	0.97 ± 0.10
PES	0.95 ± 0.04	1.08 ± 0.13	1.2 ± 0.09	1.21 ± 0.11	1.24 ± 0.07*	1.12 ± 0.07
PES after cathodal tDCS	1.02 ± 0.05	0.83 ± 0.11	1.00 ± 0.14	0.99 ± 0.12	1.06 ± 0.09	0.96 ± 0.12

mean ± standard error (mV)

*P < 0.05

**P < 0.01 vs pre

## Discussion

The Experiment 1 results indicate anodal tDCS of M1, for 10 min at an intensity of 2 mA, significantly increased the MEP amplitude 10 min after the intervention. Previous studies have reported that cortical excitability increases with anodal tDCS of M1 at an intensity of 1 mA [2]. Previous research also indicated that modulation of the neuronal membrane potential depends on stimulus polarity [3], and NMDA receptors are involved in cortical excitability changes resulting from tDCS [4, 5]. In addition, the data suggest that synaptic transmission is likely to be enhanced by increased intracellular Ca^2+^ concentrations in astrocytes [6]. Conversely, recent studies have demonstrated significant inter-individual variability in response to tDCS in healthy individuals [7, 8]. Thus, consistent with previous research, the present study may indicate increased variability in the effect of tDCS and the possibility that the effect appeared 10 min after, but not 5 min after, tDCS.

No significant change in MEP amplitude was observed with PES for 10 min in this study. Previous studies reported that PES at the motor threshold intensity inducing muscle contraction significantly increases the MEP amplitude compared to the amplitude before intervention, and decreases it with stimulation intensity at the sensory threshold. [9, 11, 14]. Although the stimulation intensity of PES used in this study was above the motor threshold, no significant increase in MEP amplitude was observed. One reason for this may be the duration of stimulation. Many previous studies using PES reported that corticospinal excitability changes with interventions of more than 20 min [9–11]. Corticospinal excitability increased more with a stimulation time of 20 min than of 40 or 60 min [10], whereas PES of less than 10 min did not significantly change MEP amplitude [16, 17]. In the present study, the intervention time of PES was set to 10 min. This duration was selected to be the same as the intervention time of tDCS. Thus, it is possible that PES for 10 min might not increase corticospinal excitability.

Combining anodal tDCS and PES resulted in no significant change in MEP amplitude before and after the intervention. Rizzo et al. examined the effect of combined anodal tDCS and PES and found that there was no significant change in MEP amplitude with PES alone but, with combined anodal tDCS and PES, the MEP amplitude duration of increase was significantly longer than with anodal tDCS alone [17]. However, Schabrun et al. reported that the MEP amplitude significantly increased after anodal tDCS or PES, but not after the two interventions combined [18], which supports our current study results. In addition, applying PES during transcranial static magnetic field stimulation, which decreases the excitability of the primary somatosensory cortex, did not affect cortical excitability [34]. Additionally, simultaneous anodal tDCS and paired associative stimulation have failed to increase corticospinal excitability [35]. Paired associative stimulation is one way to induce plasticity changes in the brain by repeating paired stimulation, which synchronizes PES and TMS of the primary sensory-motor cortex for ≥20 min [36]. Notably, motor tasks executed during anodal tDCS over M1 reduced the increase in corticospinal excitability more than anodal tDCS under the resting conditions [31, 37]. This suggests that, if somatosensory input from the periphery to the cortical region occurs during an intervention that changes the cortex excitability, such as tDCS, it may inhibit cortical excitability changes. In this study, it is possible that the neural activity of the sensory-motor cortical region by PES inhibited the slight membrane potential fluctuations of cortical neurons due to tDCS. Another possible explanation is the activation of calcium dependent gating mechanisms. The gating time-course is different than that of homeostatic plasticity. Gating is thought to instantaneously occur, whereas homeostatic plasticity is thought to be activated only when two plasticity-inducing protocols are sequentially applied [22]. This implies that gating mechanisms are more likely to underlie the effects associated with concurrent intervention. However, in the present study, corticospinal excitability increased with anodal tDCS, but not with PES. As a result, we hypothesize that sensory input to the sensory-motor area from PES might inhibit the slight membrane potential fluctuation of motor cortical neurons induced by tDCS.

Experiment 2 showed that the cathodal tDCS of M1, for 10 min at an intensity of 1 mA, significantly decreased the MEP amplitude. A previous study reported that corticospinal excitability decreased with 1 mA cathodal tDCS of M1 [2]. Regarding the mechanism of the effect of cathodal tDCS, shifting of the neuronal cell membrane potential toward hyperpolarization is involved [3, 5]. This study’s results are similar to those of previous studies, and we suggest that the MEP amplitude decreased after the intervention because corticospinal excitability was reduced by cathodal tDCS.

In previous studies, the stimulus was given for 20 min [10, 14] or 30 min [9, 11] using electrical stimulation conditions (30 Hz frequency, duty cycle of 4 sec on 6 sec off, at the intensity of the motor threshold); this was similar to this study, and an increase in MEP after stimulation was observed. Also, PES at the intensity of the motor threshold for 20 min increased the MEP amplitude after the intervention compared to before the intervention. Therefore, as in previous studies, increased corticospinal excitability may have been induced.

Based on the homeostatic metaplasticity theory [20, 23], we hypothesized that, with the combined stimulation conditions of this study, PES, after the decreased excitability of M1 induced by cathodal tDCS, would increase corticospinal excitability. However, no significant change in MEP amplitude was shown after that intervention. We believe this is related to the decreased excitability of M1, induced by cathodal tDCS, which did not reduce cortical excitability sufficiently to induce homeostatic metaplasticity. In a previous study reporting that homeostatic metaplasticity was induced, the effect after prior tDCS intervention was sustained for at least 20 min [23, 24]. However, the effect of the cathodal tDCS in this study ceased immediately after intervention, so the MEP amplitude decreased immediately after starting PES following cathodal tDCS. Therefore, we believe that, in the cathodal tDCS in this study, the time during which the MEP amplitude was decreased was insufficient to increase corticospinal excitability, so the MEP amplitude did not increase after PES.

The intervention-related effects of tDCS and PES in patients with pathological conditions have been reported [38, 39]. It is difficult to directly compare patients and healthy subjects in terms of the effects of the interventions on the brain. Because the effects of the interventions will significantly vary in healthy people, further studies on healthy participants are warranted.

## Conclusions

In this study, we attempted to increase corticospinal excitability using a combination of tDCS and PES, but we found that PES during anodal tDCS inhibited tDCS’ effect. In addition, the combination of cathodal tDCS and PES did not induce homeostatic metaplasticity and increase corticospinal excitability. Overall, we conclude that, under the conditions used in this study, PES combined with tDCS did not enhance corticospinal excitability.
